# Research progress on knowledge-attitude-practice of VTE prevention in hospitalized patients: A review

**DOI:** 10.1097/MD.0000000000038714

**Published:** 2024-07-05

**Authors:** Zaichun Pu, Ping Jia, Juan Chen, Yushuang Su, Li Wang, Qin Zhang, Dan-Yang Guo

**Affiliations:** a General Surgery Department, Xindu District People’s Hospital of Chengdu, Sichuan Province, China; b School of Medicine, University of Electronic Science and Technology of China, Chengdu, Sichuan Province, China; c Sichuan Academy of Medical Sciences and Sichuan Provincial People’s Hospital, School of Medicine, University of Electronic Science and Technology of China, Chengdu, Sichuan Province, China.

**Keywords:** attitude, behavior, knowledge, prevention, review, venous thromboembolism

## Abstract

This study analyzes and summarizes the assessment tools, current situation, and influencing factors of venous thromboembolism (VTE) prevention knowledge, attitudes, and practices (KAP) among patients. This study aimed to provide a reference basis for developing targeted health education plans and intervention strategies for patients to improve their knowledge and beliefs concerning VTE prevention. This study aimed to increase the implementation rate of VTE prevention measures and ultimately reduce the incidence of VTE.The current studies found that the factors influencing knowledge, attitude, and practice of VTE prevention in hospitalized patients include demographic factors (age, sex, education level, occupation), disease-related factors (treatment stage, injury site, and wards), and other factors (receiving VTE-related knowledge education and having medical workers at home).

## 1. Introduction

Venous thromboembolism (VTE) includes deep vein thrombosis (DVT) and pulmonary thromboembolism (PTE). Once acute pulmonary embolism (PE) occurs, mild cases may have no obvious symptoms, but the pulmonary artery pressure can progressively increase. Some patients may experience chest pain, difficulty in breathing, and hypoxemia, and severe cases may result in sudden death.^[1,2]^ DVT is considered to be the world third largest silent killer after cancer and acquired immune deficiency syndrome.^[3]^ The incidence of VTE among hospitalized patients in China has increased significantly over time, with a high-risk of VTE occurrence in high-risk populations such as surgical inpatients, reaching 53.4%.^[4]^ However, 72% of VTE cases were preventable and controllable.^[5]^ Early preventive interventions for high-risk VTE populations can reduce the relative risks of DVT and PTE by 84% and 55%, respectively.^[6]^ Early diagnosis and intervention for VTE can prevent its progression to PE and reduce the mortality rates.^[7]^

Based on the theory of knowledge, attitude, and practice, patients’ mastery of VTE prevention-related knowledge and beliefs in VTE prevention significantly affect their adherence to VTE preventive measures. Therefore, enhancing hospitalized patients’ understanding, attitudes, and practices toward VTE prevention is crucial for improving their overall preventive measures. Currently, there is a lack of comprehensive discussion regarding the knowledge, attitude, and practice of VTE prevention among hospitalized patients in China. By conducting a thorough search and literature review on this topic, the key points were summarized to provide guidance for health education aimed at enhancing VTE prevention among hospitalized patients. This will ultimately contribute to improved effectiveness in preventing VTE.

## 2. Methods

This narrative review outlines the current status and influencing factors of knowledge, attitudes, and practices (KAP) regarding VTE prevention among hospitalized patients. The main databases searched for relevant scientific papers suitable for this analysis were PubMed, Science Direct, Web of Science, and CNKI. The search queries, used singly and in conjunction, are “patients,” “venous thromboembolism,” “knowledges,” “beliefs,” “behaviors,” and “KAB” (Knowledge-Attitude-Behavior).

Ethical review and approval were waived for this study due to it not directly involving human subjects (secondary research study). The study itself was a review article and did not involve human subjects and hence patient consent was waived.

### 2.1. Assessment tool for knowledge and practice of VTE prevention

At present, foreign studies only measure the knowledge dimension or behavioral compliance separately, and there is no related research on the belief dimension. There is a lack of VTE prevention knowledge, attitude, and practice questionnaires for patients, which include 3 dimensions: knowledge, attitude, and practice. The existing survey instruments were self-developed questionnaires developed by the research teams. The research team led by Cai Yanting et al^[8]^ developed the Knowledge, Attitude, and Practice Questionnaire of Inpatients’ Participation in Venous Thrombosis Prevention, which included 3 parts: venous thrombosis knowledge, participation willingness, and participation behavior. The Cronbach α coefficient for each dimension was 0.83 to 0.899, and the split-half reliability coefficient ranged from 0.754 to 0.823. The Cronbach α coefficient of the LDVT Knowledge-attitude-Practice Questionnaire for Orthopedic Patients compiled by Tong Nian was 0.883^[9]^ and the construct validity was 0.789. The scale consists of 23 items across 3 dimensions: knowledge (9 items), belief (12 items), and behavior (2 items). The Cronbach α coefficients of the KAP questionnaire for Elderly Orthopedic Inpatients compiled by Xiuwei et al^[10]^ were 0.887, 0.858 to 1.000, and 0.877, respectively. The total content validity of the Knowledge-attitude-Practice Questionnaire for Self-Prevention of Venous Thromboembolism in Stroke Patients compiled by Ding Ranran^[11]^ was 0.832. The test-retest reliability of the questionnaire was 0.853, and the Cronbach α coefficient for internal consistency reliability was 0.78. Lin Pan^[12]^ compiled a questionnaire on the knowledge, attitude, and practice of VTE prevention in neurosurgical inpatients;the Cronbach α coefficient was 0.936 and the split-half reliability coefficient was 0.830. Feng Li-qun et al^[13]^ developed a questionnaire on the knowledge, attitude, and practice status of postoperative patients with lower extremity DVT. The questionnaire demonstrated a content validity of 0.95, Cronbach α coefficient of 0.92, and test-retest reliability of 0.85. XieYan et al^[14]^ compiled a questionnaire on the Knowledge, Attitude, and Practice of VTE Prevention in Patients undergoing major orthopedic surgery. The total Cronbach α coefficient for this questionnaire was 0.887, with a knowledge dimension at0.885, an attitude dimension of 0.817, and a behavior dimension of 0.845. Cronbach α coefficient, split-half reliability, test-retest reliability, and scale-level content validity of the maternal VTE prevention knowledge, attitude, and practice scale prepared by DengChen et al^[15]^ were 0.964, 0.694, 0.799, and 0.83, respectively.

### 2.2. Status of knowledge, attitude and practice of VTE prevention in hospitalized patients

#### 2.2.1. Status of VTE prevention knowledge in hospitalized patients

Previous studies have shown that patients have a low level of knowledge of VTE prevention. Ali Al Bshabshe^[16]^ concluded that awareness and knowledge of PE and DVT in the Saudi Arabian population are lacking. The subjects investigated by Cai Yanting et al,^[8]^ Tong Nian,^[9]^ Duan Xiuwei et al,^[10]^ Xie Yan et al,^[14]^ and Li Ling et al^[17]^ were orthopedic patients. The survey results showed that the overall knowledge scores were low and the low-scoring items mostly focused on the concepts, clinical manifestations, and risk factors of VTE. Feng Liqun^[13]^ investigated postoperative patients and found that the awareness rate of VTE prevention knowledge was low, which manifested in the aspects of VTE-inducing factors, clinical manifestations, and preventive measures. HanKe et al^[18]^ reported that knowledge about VTE prevention among bedridden patients was low; however, they did not specifically describe the knowledge scores. Yun Zhihong et al^[19]^ investigated bedridden patients and found that their knowledge of VTE prevention was above average. Ding Ranran et al^[11]^ and LinPan^[12]^ investigated stroke and neurosurgery patients, respectively, revealing that their understanding of VTE prevention was low. The scores for concepts, clinical manifestations, and risk factors of VTE were also low, and the results of studies investigating patients with stroke and neurosurgery were more consistent than those of studies investigating patients with orthopedics. Patients will receive health education on VTE from medical staff after admission. There are differences in the content and methods of education in different hospitals, but the goal remains to enhance the comprehension of VTE prevention and improve compliance with VTE prevention measures.VTE prevention knowledge includes: the concept and clinical manifestations of VTE, the high-risk factors associated with VTE, understanding the potential harm of VTE, the harm of VTE and recognizing the benefits of VTE prevention. This knowledge belongs to conceptual content and requires remembering more words. If the patient only listens to the medical staff explanation once, they may only remember part of the content, resulting in a less than ideal score when completing the questionnaire. According to patients’ level of understanding, medical staff can enhance the methods, frequency, and content of health education to improve their knowledge acquisition.

#### 2.2.2. Status of in-patient VTE prevention attitude and belief

VTE prevention attitudes and beliefs include willingness to demand, willingness to care, and willingness to make decisions. The results of various studies^[8–10,13,17]^ investigating orthopedic patients are consistent, and the belief attitude is in a moderate to positive state. A study on bedridden patients^[18,19]^ concluded that patients’ beliefs about VTE prevention were above a moderate level. LiuWei et al^[20]^ found that the health beliefs of patients with spinal cord injuries were at an upper-medium level, with the highest score for health motivation and the lowest score for health behavior disorders, which is consistent with previous studies.^[11,12]^ A study of postoperative patients^[13]^ found that patients had a high level of belief in preventing VTE, perceived the benefits of early functional exercise, and understood how to perform it. The above survey showed that patients’ beliefs and attitudes toward VTE prevention were generally positive, reflecting their favorable stance toward VTE prevention.

#### 2.2.3. Current status of VTE prevention behavior in hospitalized patients

VTE prevention includes decision making, functional exercise, physical prevention, and drug compliance. The results of previous studies^[8,13,17,19]^ were consistent. Patients have good adherence to VTE prevention measures and can continue preventive actions after receiving health education from medical staff. Xiuwei et al^[10]^ showed that VTE prevention behavior among elderly orthopedic patients was moderate. Patients can actively report their history of thrombosis and remind medical staff to implement preventive measures against thrombosis. The results obtained from the stroke and neurosurgery patients investigated in study^[11,12]^ were consistent with those of Duan Xiuwei et al. However, Zhang Ya^[21]^ showed that functional exercise was popular among patients after major orthopedic surgery, but patient compliance was low at only 36.5%. According to previous studies, patients’ VTE prevention behaviors vary greatly, with low, moderate, and high compliance levels.

Combining the aforementioned aspects of knowledge, beliefs, and behaviors yields outcomes that may differ from those outlined in KAP theory. As the researcher Xu Xin^[22]^ once mentioned, “People health behavior change may not follow the single path of knowledge, belief, and behavior, but can occur at any point along the way.” For example, new knowledge may directly change behavior, whereas attitude change is the result of behavioral change. Various studies have shown that patients’ VTE prevention knowledge is at a low or poor level, but not all patients’ beliefs and behaviors are at a low level. Studies^[8–12,18]^ have shown that patients’ knowledge of VTE prevention is low, but their behavior is moderate. In studies,^[13,14,17]^ patients’ knowledge of VTE prevention was at a low level, but their behavior was at an upper-middle level. Research^[21]^ showed that the willingness to participate in VTE prevention in patients undergoing major orthopedic surgery was at a high level, but the behavior to take preventive measures was at a low level. The results obtained from these studies contradict the expected conclusions. A possible reason for this result is that there is no standardized questionnaire available to assess the current status of KAP regarding VTE prevention in clinical settings. Although the questionnaires in each study demonstrated good reliability and validity, some were not standardized. When constructing questions related to the knowledge dimension, they are designed solely based on the literature and guidelines, and most knowledge is not experienced by patients. The scores on the questions were low, but the patient existing knowledge was sufficient to motivate action, leading to improved behavior. The bedridden patients investigated in this study^[19]^ had a moderate level of knowledge and behavior related to VTE prevention, which aligns with the theory of knowledge, attitude, and behavior. Knowledge is the foundation, belief is the driving force, behavior is the objective,and beliefs are formed through the acquisition of knowledge, leading to the adoption of VTE prevention behaviors.

## 3. Influencing factors of VTE prevention in hospitalized patients

### 3.1. Demographic factors

#### 3.1.1. Age

There are differences in knowledge of VTE prevention among patients of different ages. Previous studies^[9–11,18,19]^ have reported similar results, indicating that knowledge scores decrease with age. As individuals age, their learning abilities and memory may decline, potentially affecting their retention of knowledge regarding VTE prevention. China has become an aging society; therefore, it needs to invest more energy and patience in providing health education services for the elderly. Clinicians should educate the patients and their families to enhance their understanding of this information. The results of previous studies^[9,10,18,19]^ show that patient compliance with behavior decreases with age. This is because, as patients age, their physical function declines, they experience greater psychological pressure, and worry about their slow recovery from disease. This led to lower self-efficacy and compliance with preventive measures. Medical staff should enhance the supervision of elderly patients to encourage preventive behaviors, pay attention to the psychology of elderly patients, offer timely psychological care, and enhance patient compliance with recommended behaviors.

#### 3.1.2. Gender

In most studies, there was no significant difference in knowledge, beliefs, and practices of VTE prevention based on sex. In the study by Xie Yan et al,^[14]^ female patients exhibited a higher level of knowledge regarding VTE prevention compared to male patients. Additionally, they held more positive beliefs and were more proactive in implementing preventive measures. In an investigation of KAP related to VTE prevention, only one study found that sex was a significant influencing factor. Further studies are needed to explore whether sex is an influencing factor in the KAP regarding VTE prevention.

#### 3.1.3. Degree of education

Patients’ educational level significantly influences their understanding of and interest in acquiring knowledge, as evidenced by several studies.^[8–11,14,18,19]^ Research findings have consistently indicated that individuals with higher educational levels tend to have more knowledge and adhere better to VTE prevention measures, whereas those with lower educational levels show an opposite trend. This difference may be attributed to enhanced learning capabilities, a broader knowledge base, and heightened curiosity about learning among individuals with higher educational levels. Consequently, such patients are more adept at comprehending and retaining the information provided by healthcare professionals. Moreover, individuals with higher levels of education are more likely to appreciate and internalize the educational content provided by medical staff. Their profound understanding of the disease enables them to interact actively with healthcare providers, thus facilitating proactive measures for VTE prevention. Conversely, patients with lower educational levels often exhibit deficiencies in both knowledge and preventive behaviors. In response, healthcare professionals should customize educational interventions to meet the needs of these patients and engage their family members in the educational process to promote adherence to VTE prevention strategies, ultimately aiming to decrease the incidence of VTE.

#### 3.1.4. Occupation

Occupation plays a significant role in shaping the KAP related to VTE prevention. Various occupational groups operate within distinct social spheres, thereby influencing access to information. Studies by Cai Yanting et al^[8]^ and Tong Nian^[9]^ revealed that individuals employed as civil servants or within public institutions exhibited higher levels of knowledge in preventing VTE than workers and farmers, who scored lower. Han Ke et al^[18]^ highlighted that individuals working in administrative institutions and enterprises demonstrated the highest levels of knowledge, followed by retired individuals, workers, and farmers. Yun et al^[19]^ further indicated that workers and self-employed individuals had superior knowledge scores, followed by administrative managers and retirees, with farmers scoring lowest. While there were no significant differences in beliefs and behaviors related to VTE prevention across occupations, discrepancies in the findings may stem from variations in occupational diversity within each study. Nonetheless, it is evident that individuals with different occupational backgrounds possess varying levels of comprehension and proficiency in VTE prevention. Healthcare professionals should prioritize disseminating information and educating migrant workers using simplified language to enhance patients’ understanding of VTE prevention strategies.

### 3.2. Disease-related factors

#### 3.2.1. Treatment phase

Several studies^[8,10,18]^ have indicated that patients in the postoperative recovery phase exhibit higher scores than preoperative patients and those who are undergoing conservative treatment. Patients at various treatment stages exhibit varying levels of VTE risk and distinct susceptibilities to the disease. Additionally, there were differences in their knowledge of VTE prevention and their behavioral scores. Surgery represents an independent risk factor for VTE. After surgery, the Caprini score table was used for dynamic assessment of the patient risk level for VTE. Subsequently, health care professionals will provide VTE prevention knowledge to patients and offer additional preventive measures. Repeated health education sessions can enhance patients’ comprehension and awareness of VTE, consequently encouraging them to adopt preventive measures.

#### 3.2.2. Injury site

Among the extant studies, only Cai Yanting et al^[8]^ and Duan Xiuwei et al^[10]^ examined the variations in injury site concerning knowledge, attitude, and practice in the prevention of VTE. Both studies examined the present status of KAP regarding VTE prevention in orthopedic patients; however, they yielded divergent outcomes. A previous study demonstrated that patients with injuries at or below the waist had higher knowledge scores and better adherence to VTE prevention measures. Researchers have posited that the elevated incidence of VTE in cases of lower limb fractures and hip replacement could be attributed to the high-risk nature of these conditions. Consequently, patients exhibit increased awareness, leading to a positive reinforcement of their engagement in preventive measures. This study demonstrated that patients with injuries to the joints or ligaments of the upper limbs exhibited greater knowledge of and adherence to VTE prevention measures than those with lower limb joint or ligament injuries did. Researchers posit that patients with injuries located above the waist exhibit less severe symptoms, require shorter periods of bed rest, possess greater confidence in their recovery from the disease, and are more inclined to actively collaborate with health care professionals to implement measures to prevent VTE. Patients with lower limb injuries frequently require prolonged bed rest, and their VTE prevention behaviors are often insufficient, resulting in lower scores. The variation in outcomes could be attributed to diversity in the composition of the subjects. In a previous investigation, individuals with upper limb injuries constituted only one-third of those with lower limb injuries, whereas in a subsequent study, the distribution of patients in both groups was comparable.

#### 3.2.3. Different disease areas

In the studies conducted by Cai Yanting et al^[8]^ and HanKe et al,^[18]^ patients were sourced from various wards or departments. The participants in the previous study were from a specialized and general orthopedic ward, and the focused implementation of VTE prevention health education could substantially enhance operational efficiency, thereby intensifying the educational impact and enhancing patient adherence to preventive measures. The latter study examined bedridden patients from various medical departments, including the respiratory, neurology, neurosurgery, cardiology, and orthopedics departments. Patients from orthopedics and neurology departments exhibited higher scores than those from other medical specialities. This phenomenon could be attributed to the higher prevalence of high-risk patients in these 2 departments. Additionally, these departments have implemented more comprehensive and effective educational initiatives, leading to an enhancement in patients’ KAP related to VTE prevention. Hence, it is imperative for health care professionals in various departments to enhance the quality and techniques of health education, elevate patients’ awareness of preventive measures, and increase compliance rates with preventive protocols.

#### 3.2.4. History of thrombosis

Two studies were conducted by LinPan^[12]^ and HanKe et al^[18]^ highlighted that individuals with a history of thrombosis exhibited elevated levels of knowledge, attitude, and practice concerning VTE prevention. The variance observed in these aspects was considered to be statistically significant. Patients with a history of thrombosis were educated about VTE and received appropriate VTE treatment measures during their VTE treatment regimen. A deeper mastery of relevant knowledge leads to higher scores when responding to the questionnaire. To mitigate the risk of venous thrombosis, patients may adopt a positive attitude toward VTE prevention, leading to increased cooperation with healthcare providers and proactive engagement in relevant preventive measures.

### 3.3. Other factors

#### 3.3.1. Received VTE education

LinPan^[12]^ and XieYan et al^[14]^ highlighted that individuals who had undergone VTE education exhibited superior levels of knowledge and adherence to recommended behaviors compared with those who had not received such education. Patients who have undergone VTE education exhibit similar characteristics to patients who have experienced thrombosis. They also acquired additional information on the prevention of VTE. Initially passive, individuals retained varying degrees of knowledge. Upon understanding the detrimental effects of VTE on the human body and the benefits of VTE prevention, individuals are more likely to proactively adopt preventive measures and enhance their compliance with VTE prevention.

#### 3.3.2. There are medical workers at home

One study^[12]^ addressed the presence of medical workers at home in the initial section of the questionnaire. The findings indicated that individuals with medical professionals in VTE prevention had more KAP than those without such professionals. Patients receive VTE-related knowledge education mostly provided by the medical staff, and this part of the medical staff at home will also subtly influence patients, even in their home environment, leading to the gradual internalization of knowledge and subsequent translation into practical actions. Given that most studies did not include this factor in their questionnaires, it is plausible that additional research may have found a correlation between the presence of medical professionals in the household and their level of KAP related to VTE prevention. Further studies are required to verify this causal relationship.

## 3. Suggestion

### 3.1. Development of evaluation tools

Currently, there is a deficiency in authoritative and standardized assessment instruments for evaluating patients’ KAP regarding VTE prevention of VTE. Each researcher used a self-designed questionnaire to examine patients in a hospital setting, drawing from a single sample source. This approach may limit the generalizability of the questionnaire findings. In future research, it is recommended to select multiple hospitals during the questionnaire preparation stage. The semi-structured interview method can be employed to allow medical staff to gain insights into the specific knowledge of VTE prevention that each hospital provides to patients, including the educational methods utilized. This approach will facilitate an understanding of the extent to which patients are exposed. Subsequently, the interview data were compiled and condensed to establish the primary structure of the questionnaire. It is essential to select participants from various hospitals to guarantee the representativeness of the sample, and thereby create an assessment tool with broad applicability.

### 3.2. Design the education implementation list

The implementation sheet was developed based on the content of the VTE prevention education. The implementation sheet was used in clinical practice to assess the patients’ proficiency in VTE prevention knowledge. If the patients did not comprehend any aspect, they required further education and the effectiveness of implementing preventive measures was assessed. According to a literature analysis, current medical practices focus on providing health education to patients to prevent VTE. However, this approach predominantly involves a one-sided knowledge indoctrination. A previous study^[23]^ demonstrated that employing a health education implementation sheet to assess patients’ understanding of health education information delivered by healthcare providers can enhance their comprehension of the material, which can lead to increased adherence to preventive measures. Hence, hospitals should create customized health education implementation sheets based on the specific educational contents of their respective institutions. To encourage patients to proactively engage in preventive measures for VTE, thereby decreasing the occurrence of VTE.

## 4. Conclusion

Although VTE presents with a substantial risk, it is a preventable and controllable complication. Early preventive intervention for patients at a high-risk of VTE has the potential to decrease the relative risk of DVT by 84% and PTE by 55%.^[6]^ In recent years, China has engaged in proactive efforts to enhance the standardization of VTE prevention and management. Most hospitals adhere to a standardized system for VTE prevention and treatment; however, awareness of VTE prevention in inpatients remains low and requires further enhancement. There are still numerous shortcomings in the attitudes and behaviors toward prevention. Hence, enhancing the knowledge, attitude, and practice of VTE prevention in hospitalized patients is crucial to promote adherence to VTE prevention protocols. Howere, current studies have not conducted a comprehensive analysis of the factors that influence patients’ KAP regarding VTE prevention. In conclusion, most influencing factors stem from the demographic and disease-related characteristics of the patients, whereas other potential influencing factors have not been thoroughly investigated. Knowledge, attitude, and behavior regarding VTE prevention are frequently influenced by various factors.^[24]^ For instance, further clarification is required regarding the influence of patients’ psychological factors and social support on their knowledge, attitude, and practice regarding VTE prevention. Existing research on patients’ KAP regarding VTE prevention has focused primarily on status surveys. However, there is a gap in the literature as no study has investigated the potential correlation between patients’ KAP related to VTE and the actual incidence of VTE. Hence, future research should systematically investigate the association between the present state of KAP regarding VTE prevention, and the occurrence of VTE. This could facilitate the integration of the knowledge-attitude-practice theory into health education interventions aimed at preventing VTE.

## Author contributions

**Conceptualization:** Zaichun Pu.

**Supervision:** Ping Jia.

**Writing – original draft:** Zaichun Pu, Juan Chen.

**Writing – review & editing:** Yushuang Su, Li Wang, Qin Zhang, Dan-Yang Guo.
